# Finite element analysis of a new preoperative traction for cervical kyphosis: suspensory traction

**DOI:** 10.1007/s11517-024-03113-z

**Published:** 2024-05-06

**Authors:** Hongyu Chen, Tianchi Wu, Shengfa Pan, Li Zhang, Yanbin Zhao, Xin Chen, Yu Sun, William W. Lu, Feifei Zhou

**Affiliations:** 1 Department of Orthopaedics, Peking University Third Hospital, Beijing, China; 2 Engineering Research Center of Bone and Joint Precision Medicine, Beijing, China; 3 Beijing Key Laboratory of Spinal Disease Research, Beijing, China; 4 Department of Orthopaedics and Traumatology, The University of Hong Kong, Hong Kong, Hong Kong SAR China; 5 Shenzhen Institutes of Advanced Technology, Chinese Academy of Science, Shenzhen, China

**Keywords:** Preoperative traction, Suspensory traction, Finite element analysis, Cervical kyphosis, Intervertebral disc, Ligament

## Abstract

A finite element model of cervical kyphosis was established to analyze the stress of cervical spine under suspensory traction and to explore the mechanism and effect of it. A patient with typical cervical kyphosis (C2-C5) underwent CT scan imaging, and 3D slicer was used to reconstruct the C2 to T2 vertebral bodies. The reconstructed data was imported into Hypermesh 2020 and Abaqus 2017 for meshing and finite element analysis. The changes of the kyphotic angle and the von Mises stress on the annulus fibrosus of each intervertebral disc and ligaments were analyzed under suspensory traction conditions. With the increase of suspensory traction weight, the overall kyphosis of cervical spine showed a decreasing trend. The correction of kyphosis was mainly contributed by the change of kyphotic segments. The kyphotic angle of C2-C5 was corrected from 45° to 13° finally. In cervical intervertebral discs, the stress was concentrated to anterior and posterior part, except for C4-5. The stress of the anterior longitudinal ligament (ALL) decreased from the rostral to the caudal, and the high level von Mises stress of the kyphotic segments appeared at C2-C3, C3-C4, and C4-C5. The roles of the other ligaments were not obvious. The kyphotic angle was significantly reduced by the suspensory traction. Shear effect due to the high von Mises stress in the anterior and posterior parts of annulus fibrosus and the tension on the anterior longitudinal ligament play a role in the correction of cervical kyphosis.

**Graphical Abstract:**

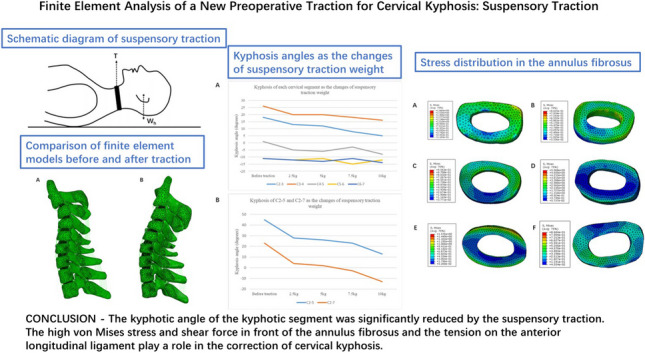

## Introduction

The treatment of cervical kyphosis is one of difficult problems in spinal surgery. Surgical intervention should be considered for patients suffering from progressive deformity, myelopathy, radiculopathy, or dysfunction who do not respond to conservative treatment [1–3]. In patients with moderate or severe cervical kyphosis, contracture of surrounding soft tissue, loss of neurons, and demyelination of nerve fibers caused by mechanical pressure and vascular compression lead to great surgical difficulty [4–6]. Some scholars have proposed that there are benefits of cervical traction before surgery, including reducing the curvature of deformity before surgery, reducing the risk of surgery, improving the correction outcome, and reducing the incidence of surgical complications. At present, axial skull traction and Halo traction are the commonly used preoperative traction techniques. There are differences in the specific timing of use, duration of use, and traction details in different reports [7–9], but the mechanism is to apply axial traction force on the spine. Moreover, the traditional traction mode has some inevitable shortcomings. Patients have to take long-time traction that restricts daily life, perhaps even leading to complications such as structural damage or infection caused by pins and neurological deterioration.

**Fig. 1:**
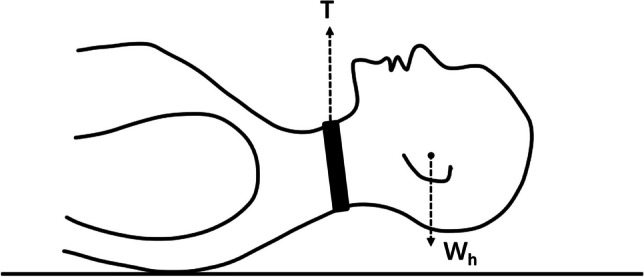
Sketch of forces on the cervical spine during suspensory traction

Upon above factors, we propose a traction mode different from traditional traction, named suspensory traction. It achieves preoperative correction by applying a traction force opposite to the kyphotic direction to cervical spine segments (Fig. 1). Satisfying correction effects have been observed in clinical practice [10], but the correction mechanism is still unclear. Finite element analysis has been widely used on spinal deformity correction, which can simulate the complex mechanical environment and measure the internal biomechanical indicators [11–13]. In the present study, a finite element model of cervical kyphosis was established, in which the state of suspensory traction was simulated, and the principle of its traction mode was explained from the perspective of biomechanics, providing theoretical basis and reference for clinical treatment.

## Method

### The procedure of suspensory traction

The patient lies flat on the bed with an approximate 10-cm-width traction harness wrapping around the neck. The gravity of the head is mostly balanced by the traction harness; the head-harness-torso could be simplified as a cantilever beam, leaving highest moment next to the harness (Fig. 1). The patient’s head does not need to be lifted off the bed surface at the beginning. When the patient adapts to the traction force, the traction weight is gradually increased. The appropriate traction weight is determined according to the body weight of the patient, and the final range is 6–12 kg. Traction is increased until the occiput and back are clear of the bed surface. Unlike skull traction, a continuous traction method, the duration of suspensory traction can be adjusted according to individual tolerance and life needs. The duration of the traction is a few minutes at the beginning and gradually extended thereafter. The total duration of the traction is more than 8 h per day.

### Establishment of finite element model

This study was approved by Peking University Third Hospital Medical Science Research Ethics Committee. The model constructed in this study was based on CT images of a 12-year-old boy. A dynamic radiograph of the cervical spine was used on him and suggested cervical kyphosis. The CT images were put into 3D slicer (http://www.slicer.org) for geometric reconstruction of the vertebral bodies from C2 to T2. The reconstructed geometry was transferred to Hypermesh 2020 (Altair Technologies, Inc., CA, USA) and Abaqus 2017 (Abaqus, Inc., Providence, RI, USA) to perform mesh work and conduct finite element (FE) analysis.

During traction, the load will not cause significant deformation of the vertebral body. Therefore, in order to simplify the model, there was no distinction between cortical bone and cancellous bone. The biomechanical behavior of bone was evaluated through a phantom-less bone mineral density (BMD) measurement (Table 1) [14]. Then, BMD was converted to Young’s modulus as per Eq. 1 [15].

**Table 1:** Individual material properties in the finite element model

	BMD (mg/cc)	Young’s modulus (MPa)	Poisson’s ratio
C2	200.4	403.10	0.30
C3	242.1	589.43	
C4	319.2	1,027.46	
C5	301.3	914.93	
C6	262.6	694.04	
C7	229.1	527.53	
T1	165.7	275.07	
T2	187.4	352.26	
Nucleus pulposus	——	1.0	0.49
Anulus fibrosus	——	3.4	0.40

1 $$\left\{\begin{array}{ll}E=0001& (BMD=0.00)\\ E=33900\times {(\frac{BMD}{1000})}^{2.2}& (0.00 < BMD < 0.27mg/cc)\\ E=5407\times BMD+469& 0.27\le BMD\le -6-mg/cc\\ E=10200\times {(\frac{BMD}{1000})}^{2.01}& 0.60mg/cc\le BMD\end{array}\right.$$

The intervertebral disc was added to the space between adjacent vertebral bodies, which was divided into two parts, the nucleus pulposus and the annulus fibrosus. The nucleus pulposus structure covered 25–50% of the surface area of the upper and lower vertebral bodies and 40–50% of the volume of the whole intervertebral disc [16–21]. The reconstructed disc was attached to the upper and lower surfaces of the adjacent vertebral bodies with all translational and rotational degrees of freedom being the same. To reduce the nonlinearity of the finite element model, linear elastic mechanical properties of the nucleus and annulus were defined.

**Fig. 2:**
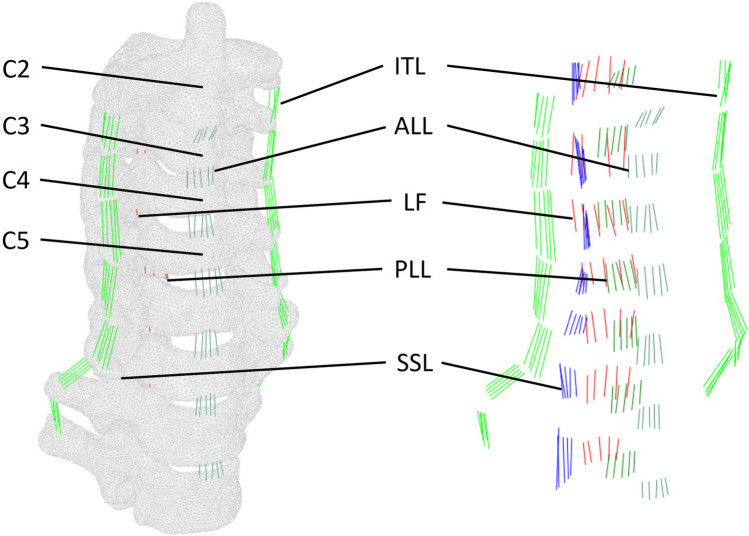
Anatomical schematics of vertebral bodies, ligaments, and truss elements in the FEA model

Several truss elements were utilized to represent the major ligament structures (Table 2), including posterior longitudinal ligament (PLL), anterior longitudinal ligament (ALL), interspinous ligament (ISL), supraspinous ligament (SSL), intertransverse ligament (ITL), and ligamentum flavum (LF) (Fig. 2). Only one element was mapped for each filament in every ligament (T3D2), where only tension loads are active, and there is no moment transfer. In order to simplify the processing and avoid inaccurate muscle strength, the FE model ignored all the muscles of the cervical and cervicothoracic segments.

**Table 2:** Cross-sectional area and mechanical properties of each ligament

	Young’s modulus (MPa)	Poisson’s ratio	Cross-section area (mm2)
Anterior longitudinal ligament (ALL)	20	0.3	38
Posterior longitudinal ligament (PLL)	70		20
Interspinal ligament (ISL)	28		35.5
Supraspinal ligament (SSL)	28		35.5
Intertransverse ligament (ITL)	50		10
Ligamentum flavum (LF)	50		60

### Boundary and loading conditions

The bottom surface of the lower endplate of T2 was fully fixed in all degrees of freedom, and other vertebrae were not constrained. Since geometrical collision was detected in the FE analysis of pre-study, Coulomb friction was applied between posterior parts of all vertebral bodies; friction coefficient was set 0.2, and no vertical penetration was allowed between master and slave surface. The patient’s head weight was estimated to be 61.4 N based on the average head weight of 7.83% of body weight reported in the literature [22]. In the present FE model, the gravity load of the head is applied to the C2 segment through structural distribution, and the change in the magnitude of the moment caused by the moment offset is corrected. The force arm length was obtained by measuring the distance between the center of mass of the head and the center of C2 vertebral lamina under lateral X-ray film in traction state. The length of the force arm in the present FE model is 65 mm. The traction force was applied uniformly behind the C3 vertebral lamina to simulate the force on the neck during suspensory traction. Considering that it is difficult to choose a suitable location for the force of the neck gravity, and the Resultant moment is relatively small (soft tissue is distributed around the vertebral bodies, the moments in different directions cancel each other out), the gravity of the neck is not added.

### Assessment indices

The kyphotic angle is defined as the included angle between the uppermost and lowermost vertebral inferior endplate of kyphosis segment, which is used to evaluate the degree of kyphosis. According to the usual maximum traction weight, 10 kg, we divide it into four parts to explore the appropriate initial traction weight and the change of correction effect with the increase of traction weight. The magnitude of the kyphotic angle of the lower cervical spine was analyzed before traction and under four different traction forces (2.5 kg, 5 kg, 7.5 kg, 10 kg). The angle is defined as a positive value for kyphosis and a negative value for lordosis. The von Mises stress on the annulus fibrosus of each intervertebral disc and ligaments was analyzed under the maximum traction force (10 kg).

### Validation

The construction of FE model was same as our previous-published FE study [23]. The FE model was validated against the post-stretch X-ray image (Table 3). The error of Cobb angle between the FE model and the X-ray image was caused by the estimated material properties of soft tissues, since the surrounding ligaments work together to keep the dynamic stability of the cervical spine—estimated material properties might lead to a slightly different stable position compared with the realistic outcomes. The FE model still obtained a comparable magnitude of the Cobb angle, allowing us to believe the simulation is reliable and reasonable.

**Table 3:** Comparison of Cobb angle of each cervical segment between FE model and X-ray image

Cervical segment	FE model (degrees)	X-ray image (degrees)
C2-3	− 5	− 6.3
C3-4	− 15	− 11.2
C4-5	8	8.3
C5-6	12	9.5
C6-7	14	11.7

Cobb angle is positive while lordosis

## Results

### Kyphotic angle

**Fig. 3:**
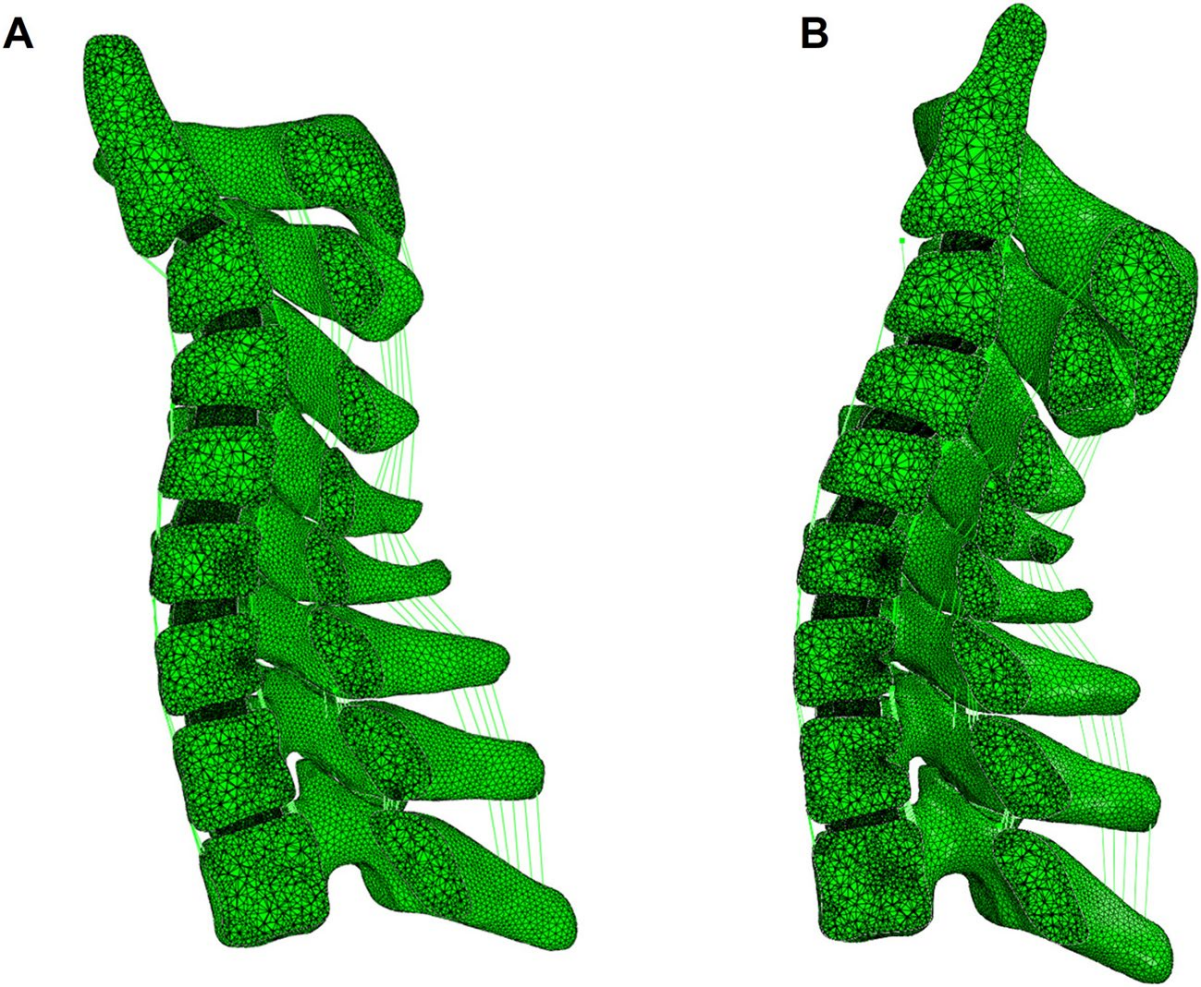
Sagittal cross-sectional view of the finite element model. **A** Before traction; **B** the traction force reached the maximum value

Kyphotic angle of each cervical segment before traction and under four different traction forces is listed in the table as follows (Table 4). The kyphotic angle of C2-5 was corrected from 45° to 13° finally, with a correction rate of 71.1% (Fig. 3).

**Table 4:** Kyphotic angle of each cervical segment under different suspensory traction forces

	Before traction (degrees)	Weight of suspensory traction			
		2.5 kg (degrees)	5 kg (degrees)	7.5 kg (degrees)	10 kg (degrees)
C2-3	18	13	12	8	5
C3-4	26	20	20	18	15
C4-5	1	− 5	− 6	− 3	− 8
C5-6	− 11	− 12	− 11	− 15	− 12
C6-7	− 11	− 12	− 13	− 11	− 14
C2-5	45	28	26	23	13
C2-7	23	4	2	-3	-13

Kyphotic angle is positive while lordosis angle negative

**Fig. 4:**
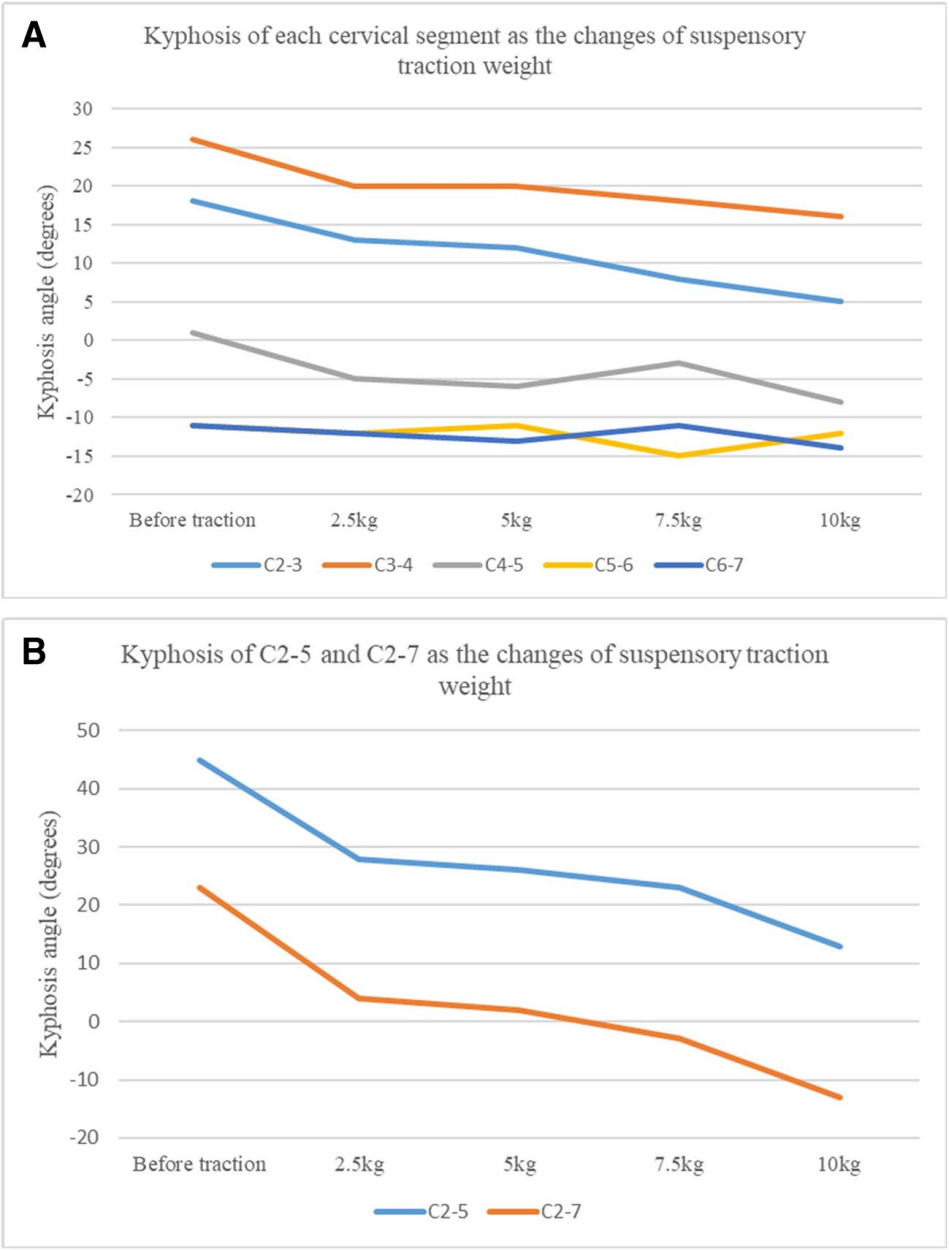
Kyphosis of cervical segment as the changes of suspensory traction weight. **A** Each cervical segment; **B** C2-5 and C2-7

The kyphotic angles of the segments located in the kyphosis segments (C2-3, C3-4, and C4-5) decreased with the increase of suspensory traction weight, while the angles of the other cervical segments (C5-6 and C6-7) did not change significantly with the rise of suspensory traction weight (Fig. 4). The kyphotic angles of the kyphotic segments and the whole cervical spine dropped significantly with the increase of the suspensory traction weight. Moreover, the kyphotic angles showed a dramatically downward trend. When the traction was initially applied, same when the traction force was close to maximum.

### Stress of intervertebral discs

**Fig. 5:**
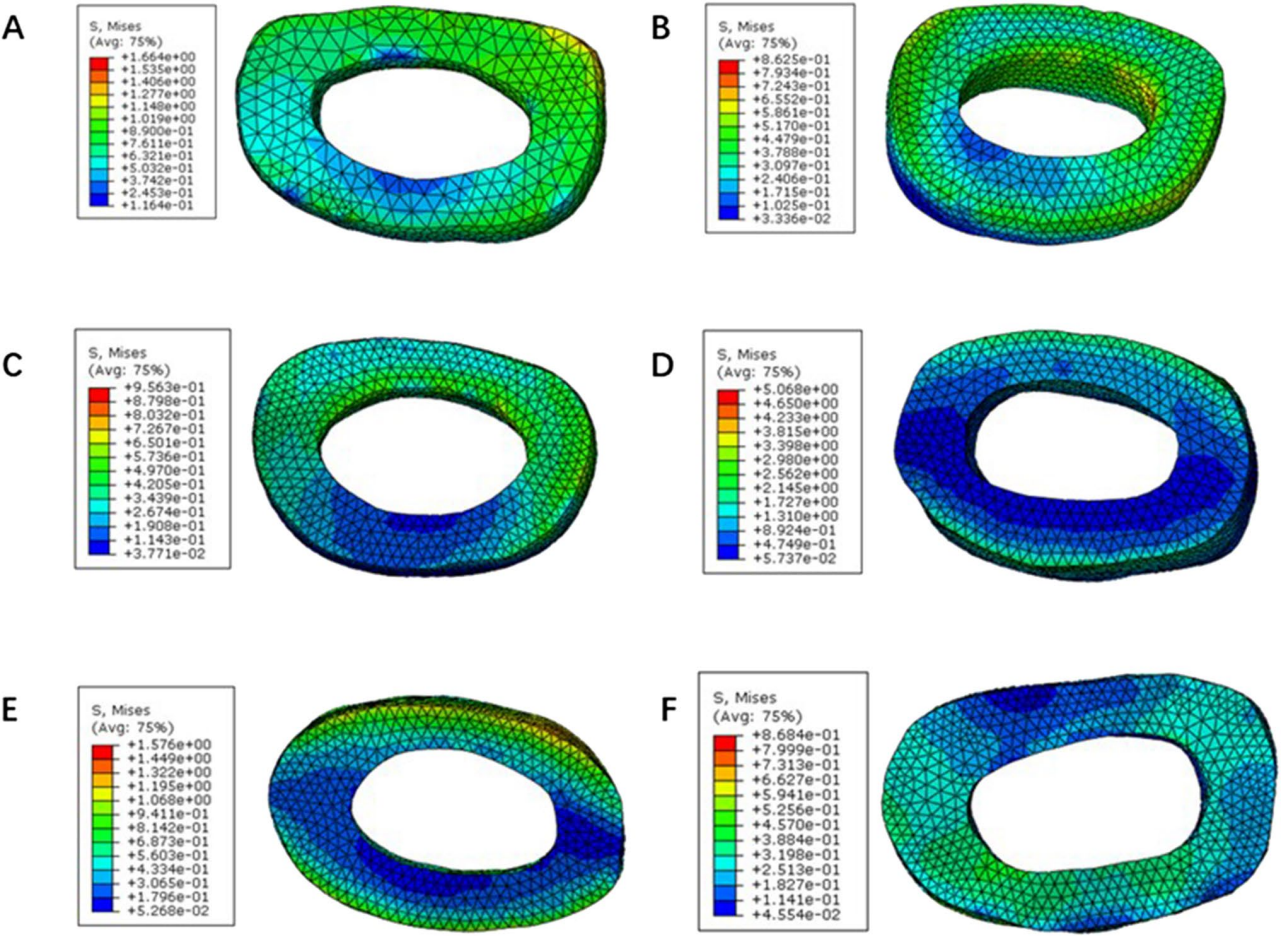
Stress distribution in the annulus fibrosus. **A** C2-3; **B** C3-4; **C** C4-5; **D** C5-6; **E** C6-7; **F** C7-T1

Stress pattern of C2-3 showed that the posterior part was subjected to a larger force compared with anterior part; C3-4 exhibited nearly same stress distribution even though C2-3 had higher stress level than C3-4 (Fig. 5). Stress of C4-5 concentrated to lateral and posterior part, but the entire intervertebral disc was at low stress state; however, stress localized to anterior and posterior zone in C5-6 and C6-C7, indicating two long and narrow parts deformed largely. C7-T1 exhibited same stress level as the C4-5.

### Stress of ligaments

**Fig. 6:**
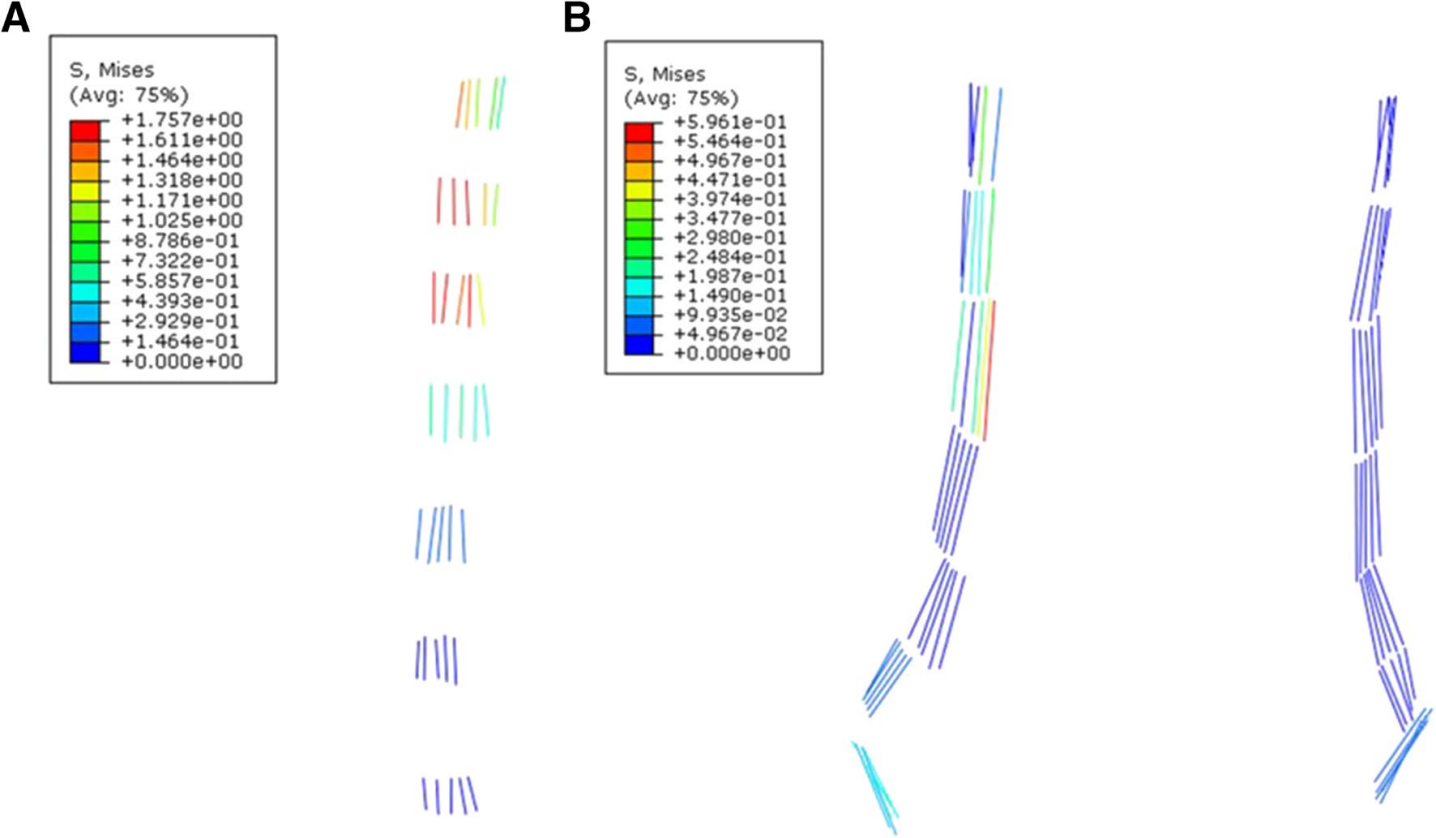
Stress distribution in ligament. **A** ALL; **B** ITL

The stress of the anterior longitudinal ligament (ALL) decreased from the rostral to the caudal (Fig. 6), and the high level von Mises stress of the kyphotic segments appeared at C2-C3, C3-C4, and C4-C5, ranging from 1.76 to 1.03 MPa in each filament, corresponding to the large backward flexion (Fig. 3). The intertransverse ligament (ITL) did not play a significant role in suspension traction. The tension of posterior longitudinal ligament (PLL), interspinous ligament (ISL), and ligamentum flavum (LF) was almost zero.

## Discussion

### Advantages of suspensory traction

Preoperative traction helps relax anterior soft tissues, making it easy to open anterior structures in surgery, which provides safe partial correction and typically decreases the amount of corrective force that needs to be applied to the cervical spine [24, 25]. Skull traction and Halo traction require stable force points and therefore require pin insertion, which carries the risk of infection or blood vessel damage. If the wounds are not properly cared for during halo traction, it may even lead to osteomyelitis and intracranial abscesses [26], which are severe and difficult to deal with. Suspensory traction relies on the traction belt behind the cervical spine to exert a vertical upward traction force, which is a non-invasive operation without the above complications.

Lim et al. reported a patient with vertebral fracture due to Halo gravity traction [27]. For elderly patients with osteoporosis, the traditional traction method may lead to iatrogenic fracture because of the large traction force. In addition, the direction of the traction force of Halo traction is parallel to the spine, which may be directly applied to the nerve and cause nerve function damage. Damage to cranial nerves and brachial plexus caused by Halo traction has been reported [28–30]. Suspensory traction mainly relies on the moment generated by the head’s own gravity in the opposite direction of the kyphosis to perform correction. It exerts less force on the cervical spine than conventional traction. In addition, the kyphosis of the cervical spine will be improved after traction, thus reducing the direct pressure on the spinal cord and the high tension of the spinal cord caused by kyphosis.

The quality of life of patients is also a part to be considered in the treatment. The traditional skull traction is continuous traction, which will cause trouble for patients to eat, bathe, etc. Suspensory traction is easy to install and remove, and it is intermittent traction, so the patient can adjust the traction conveniently. In addition, the sleep quality of patients is improved because no traction is required during sleep at night, which is no different from normal life.

In previously reported cases, suspensory traction has demonstrated its good correction effect and safety. For patients with severe cervical kyphosis, suspensory traction can reduce the kyphosis angle (the angle between the lower or posterior edge of the vertebral body at the upper and lower ends of kyphosis) before surgery by 66.7°, contributing 86% to the total correction effect [31], which reflects the effectiveness of this traction method. A patient with neurofibromatosis (NF-1) developed quadriplegia after skull traction and suspensory traction combined with cervical spondylolysis were performed on him. Then, the patient’s kyphotic angle significantly reduced from 125° to 30°, and the neural function gradually recovered in the follow-up without complications.

### Construction of suspensory traction FE model

Previous studies have demonstrated the effectiveness of suspensory traction solely through clinical observations, without delving into its underlying mechanism [31]. In contrast, the present study offers a biomechanical explanation by employing finite element analysis. When constructing the finite element model, we initially set no additional conditions between the posterior structures of the cervical spine. However, when the FE analysis was performed, there was a large difference in the kyphosis angle of the cervical spine between the model (the kyphosis angle of C2-5: 26°) and the data actually measured on the lateral cervical spine radiograph during traction (the kyphosis angle of C2-5: 7°). When looking for the cause, it was found that the bones behind the spinal canal overlapped to a certain extent. Therefore, rules for collisions between bones are added to the model. The cervical kyphosis angle of the improved model (the kyphosis angle of C2-5: 13°) was different from that of the initial model, which proved that the compression between the bones did exist.

In previous studies, the use of FE analysis did not mention imposing bone-to-bone collision conditions on the model [32–35]. An interesting finding of the present study is that in the extension position of the cervical spine, the bones behind the spinal cord may contact and affect the results of FE analysis. Therefore, it is suggested that scholars should consider the compression between bones during cervical motion when performing FE analysis of the cervical spine.

### Mechanism of suspensory traction

With the increase of the weight of suspensory traction, the condition of cervical kyphosis was improved, especially the kyphotic segments, which reflected the pertinence of suspensory traction for the correction of kyphosis. The study demonstrated that the corrective effect of suspensory traction increased with the rise of traction force. The current research analyzed the condition of cervical spine under different traction weight. Before the surgery, this traction will be removed, but its traction effect will be maintained to a large extent. This is attributed to the compliance of the soft tissue contractures anterior to the cervical spine under high stress. During the surgery, the cervical spine will be further corrected and fixed to maintain the correction effect.

**Fig. 7:**
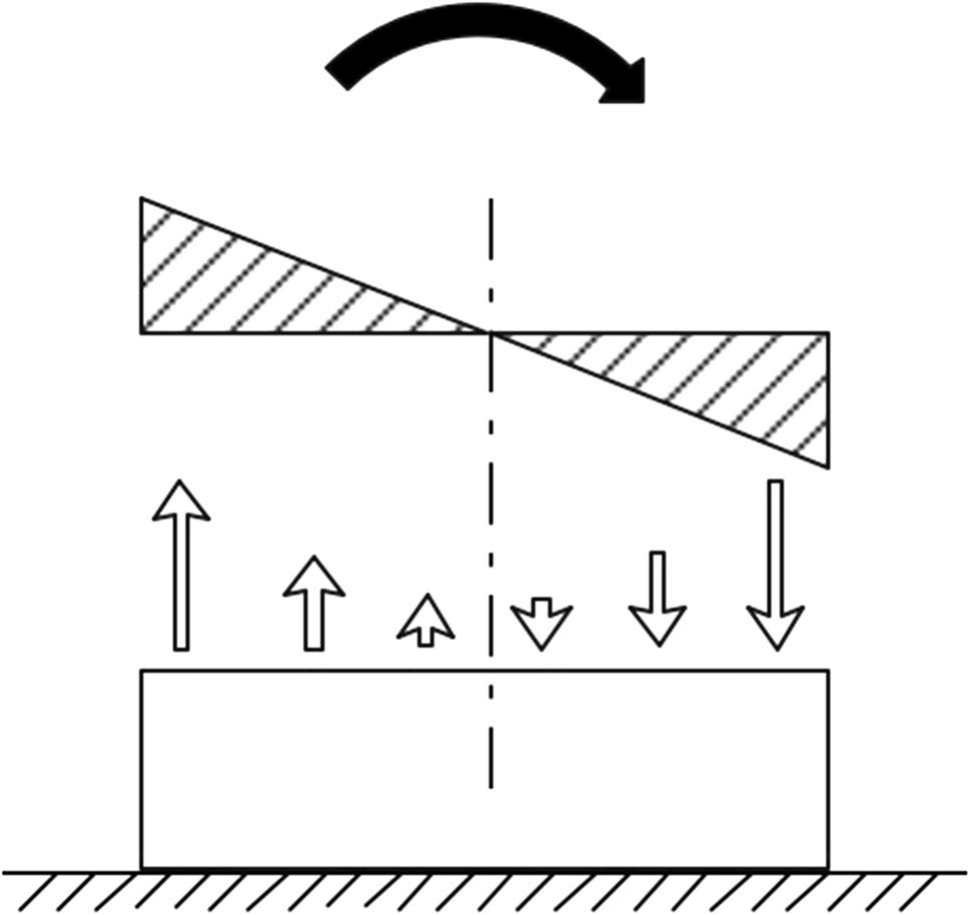
The 2D stress schematic of intervertebral disc. A moment was applied onto one end of a long beam of which the shear effect is neglected. The normal stress in the left and right side exhibits tensile and compress behavior respectively

The head gravity created a bending moment effect in the kyphosis segments. Due to the component of gravity was mainly perpendicular to the vertebral axis, the compression of vertebral bodies in axial direction did not change significantly. Generally speaking, the stress level in all intervertebral discs was pretty low, probably causing by the loading condition. The compression—component of the head weight—was small since the angle between gravity and the caudal-cephalad axis of the C2 was below 10° (i.e., sinuous value no more than 0.2). Besides, the induced moment intended to flex the cervical towards posterior, resulting in anterior and posterior parts subjected to relatively high stress (Fig. 7). The stress mode produces a shearing effect.

In addition to the bending moment, the intervertebral discs were also subjected to an axial pressure, so the stress on the anterior annulus of the kyphotic segments was slightly less than the posterior. When the traction force was close to the maximum value, the lamina and spinous collided, causing the center of relative rotation between the vertebral bodies to move backward from the facet joint and nucleus pulposus area to the collision area. The rearward shift of the center of rotation produced a larger degree of eccentric motion, which explained why the kyphosis angle drops significantly as the traction force approached the maximum.

Wang et al. [32] analyzed the biomechanical characteristics of conventional traction with and without posterior cervical support and found that the posterior annulus stress was also greater than the anterior. However, the stress of anterior annulus fibrosus of some segments was very small, even almost zero, which was not helpful for the correction of kyphosis. In axial traction, PLL, LF, and ISL of middle cervical spine received greater tension, while the ALL experienced a very low tensile force [32]. In contrast, the ALLs in our finite element model were subjected to a large amount of tension, which is a mainly responsible ligament that limits the excessive extension of the cervical spine, thus allowing suspensory traction to produce a corrective kyphotic effect.

## Limitations

The anterior and posterior longitudinal ligaments of the cervical spine were added to the FE model. However, there are no reported ligament parameters in patients with cervical kyphosis to the authors’ knowledge, so the ligaments in this model were assigned to normal population data. The same problem appears in the material properties of intervertebral discs. Considering that cervical kyphosis will lead to the shortening of the anterior structure of the cervical spine and the extension or even elasticity losing of the posterior structure, there will be some errors between the model and the actual situation.

In addition, this model lacks the corresponding creep material constitution of soft tissues such as neck muscles, so it cannot analyze the relaxation effect of anterior muscles brought by long-term traction, but it can predict the maximum traction effect. Because the muscle and other soft tissues will deform and rebound after the external load of traction is removed, the result of the present FE analysis is the ideal correction effect.

The biomechanical model of the present study is based on the CT of C2-C5 cervical kyphosis, so the conclusion is only applicable to the case of cervical kyphosis at this position. The optimal traction position for different segments of kyphosis needs further study.

## Conclusion

The present finite element analysis demonstrated that the new preoperative traction technique, suspensory traction, can effectively reduce the degree of cervical kyphosis. The mechanism of this correction came from the moment generated by the head gravity, which acted on the annulus fibrosus and the anterior longitudinal ligament to form strain. Another finding of this study is that there will be contact and force transmission between the lamina and spinous of the cervical spine during correction.
